# 

**Published:** 1921-06

**Authors:** 


					CONTENTS. page
s
^Orrie Problems in Surgical Treatment of Abdominal Conditions.
By Forbes Fraser, C.B.E., F.R.C.S.
Th
e Present Position of Psychotherapy.
By R. G. Gordon, M.D.,
B.Sc., M.R.C.P.Ed.
Teething Myth.
By R. C. Clarke, O.B.E., M.B., B.Cli. Brist.
M.R.C.P.
28
Egress of the Medical Sciences :
Medicine.
Cecil Clarke, M.B., B.S. Lond. ...
37
Su
rgery.
James Swain, C.B., C.B.E., M.S., M.D.
43
Gynaecology.
R. S. S. Statham, O.B.E., M.D. Brist.
47
eviews of Books
49
^'torial Notes
58
Library of the Bristol Medico-Chirurgical Society 64
eetings of Societies
66
k'tuar
y =
John Matthew Fortescue-Brickdale
6g
?cal Medical Notes
72

				

